# First experiences with the introduction of genetic counselors in human genetic services in the German‐speaking countries

**DOI:** 10.1002/jgc4.1979

**Published:** 2024-11-11

**Authors:** Simone Heidemann, Johannes Zschocke, Gunda Schwaninger

**Affiliations:** ^1^ Institute of Tumor Genetics North Kiel Germany; ^2^ Institute of Human Genetics Medical University of Innsbruck Innsbruck Austria

**Keywords:** Austria, Germany, Switzerland, genetic counselors, medical genetic services, professional development, service delivery models

## Abstract

In 2019, the Medical University of Innsbruck introduced the first Genetic and Genomic Counseling master's program in the German‐speaking countries. A major challenge of this process was the absence of practicing Genetic Counselors (GC) in these countries, leading to a lack of experience with GCs in medical genetic services and the absence of a legal framework for the profession. Consequently, student placements within the program commenced with neither the students nor their supervising consultants having any previous experience of collaborative teamwork between clinical geneticists and GCs. To share insights from the initial implementation phase, supervising consultants from the seven student placement institutes were invited to participate in semi‐structured interviews guided by open‐ended questions. From these interviews, three models of interprofessional teamwork between GCs and clinical geneticists emerged: (1) the alternating or tandem model, (2) qualified preliminary and follow‐up patient interviews, and (3) the provision of genetic (counseling) services without genetic counseling in the legal sense. In addition, the interviewees provided recommendations for addressing legal concerns and for the effective establishment of appropriate compensation structures for GCs within the German‐speaking countries. Clinical geneticists taking part in the study estimated that the integration of GCs could potentially enhance their counseling capacity by as much as 50%. Importantly, they did not foresee any reduction in counseling quality caused by the inclusion of GCs. This study provides evidence that the GC profession can provide additional skills to human genetic services and positively impact both patient support and overall capacity of genetic services also in the German‐speaking countries.

**What is known about the topic**

While the profession of GCs has achieved recognition across Europe and worldwide, there remain several European nations in which GCs have not been integrated into the public health systems. Notably, this group includes Germany, Austria, and the German‐speaking region of Switzerland.

**What this paper adds to the topic**

The inception of the initial Genetic Counseling master's program in Austria took place in 2019, leading to the graduation of the first seven GCs educated in the region in 2022. Drawing insights from the students' clinical training experiences, we elucidate models for integrating the profession into pre‐existing medical genetic services across Germany, Austria, and the German‐speaking regions of Switzerland.

## 1 INTRODUCTION

The initial university program for Genetic Counselors (GCs) was initiated in 1969 at Sarah Laurance College in the US (Heimler, 1997) and it took half a century for the first German‐taught program to start educating students. Since 2019, the Medical University of Innsbruck has offered a five‐semester German‐taught MSc program in Genetic and Genomic Counseling accredited by the European Board of Medical Genetics (EBMG) (Skirton et al., 2013). The program follows the international curriculum developed by the EBMG and encourages graduates to register as European certified GCs allowing them to practice across many European countries. The content focuses on evidence‐based genetics knowledge, communication and counseling skills, ethical and legal underpinning of the profession, and a structured practical placement in a genetic service (Schwaninger et al., 2021). In contrast to numerous European nations, the GC profession has yet to be established in Germany, Austria and the German‐speaking part of Switzerland (Abacan et al., 2019; Catapano et al., 2022; Ormond et al., 2018).

Genetic Counselors play a crucial role in helping individuals in comprehending and adjusting to the medical, psychological, and familial consequences of genetic conditions, while also guiding them through the intricacies of genetic testing. To achieve this, they collect and interpret medical histories to evaluate the likelihood of disease occurrence or recurrence. They educate those seeking counseling about inheritance patterns, testing procedures, management strategies, preventive measures, and available resources, empowering them to make well‐informed choices (Resta et al., 2006). A significant aspect of their role involves offering emotional support to both patients and their families. Therefore, GCs receive more intensive training in patient‐centered communication and psychosocial theories than commonly provided in medical specialist training.

A comprehensive international analysis involving 189 GCs from 22 countries has demonstrated a quite consistent scope of clinical practice among GCs (Ormond et al., 2023). Core tasks encompass case preparation, establishing rapport and outlining expectations, conducting family and medical history assessments, offering risk evaluation and counseling, delivering foundational genetic knowledge to patients, discussing test options, potential outcomes, and ramifications, which includes offering management recommendations based on test results. Additionally, GCs aid individuals in making informed decisions and conducting psychosocial assessments to provide supplementary support. Interestingly, when surveyed, tasks within the medical history category, such as physical measurements and diagnoses, were found to be performed least (Catapano et al., 2022; Ormond et al., 2023).

In the early days, clinical genetic services in **Western Germany** were primarily delivered by pediatricians and clinicians who underwent an additional 2‐year training to acquire the supplementary designation of “Medical Genetics”. “In contrast, East Germany already established human genetics as a specialty in the 1970s”. Following the reunification of West and East Germany human genetics emerged as an autonomous medical discipline for the unified nation around 1994. During this period, collaborations between physicians and clinical laboratory scientists as well as social workers were commonplace within the realm of genetic counseling. In 2010, the German Diagnostic Act (GenDG) was introduced – among other topics – for genetic investigations for medical purposes (Bundesrat, 2011). Following the recommendations of the Genetic Diagnostic Commission (GEKO, 2011) this law restricts the provision of genetic counseling to qualified physicians including specialist human geneticists and physicians with additional qualification (“Zusatzbezeichnung”) in medical genetics or special training in genetic counseling (§ 7(3) GenDG). However, with the consent of the counselee, the GenDG permits the involvement of additional expert professionals in genetic consultations (§ 10(3) GenDG). As of 2021, the German Human Genetic Board documented 388 registered certified human genetic specialists and 176 physicians holding the additional qualification (“Zusatzbezeichnung”) ‘Medical Genetics,’ working across over 100 genetic centers, serving an approximate population of 80 million citizens. It is worth noting that more than 20% of human/medical genetic specialists (MGs) are expected to retire within this decade (Schmidtke et al., 2020).

In **Austria**, clinical genetic services emerged during the 1970s, alongside the change of the penal law for termination of pregnancies in 1975 (Mayer et al., 2009; Petermann et al., 2017). In 1994, Austria introduced the Gene Technology Act (GTG), which regulates – among other topics – the use of genetic diagnostic tests, including data protection, informed consent, the inclusion of relatives, and Genetic Counseling (BMG, 2018).

In addition, an advisory board on gene technology to the Austrian Government regularly publishes the Gene Technology Book, containing up‐to‐date amendments to the GTG (Austrian Ministry of Health, 2018; Gschmeidler & Flatscher‐Thoeni, 2013).

Interestingly, the specialty of medical biology was only renamed to Medical Genetics in 2007. As of April 2020, the Austrian Medical Board reports 30 registered Medical Geneticists (MGs), most of whom work in one of the six designated Centers for Medical Genetics, serving a population of 8.8 million citizens. It is worth noting that approximately half of the MGs are anticipated to retire within the upcoming decade.

In **Switzerland**, Medical Genetics is an independent medical discipline since 1999. An independent GC profession exists in the French‐speaking western part of Switzerland; however, the GC profession is officially recognized only in the province (Kanton) Waadt/Vaud. In 2021, 11 GCs were employed in the French‐speaking western part of Switzerland. GCs practicing in Switzerland have been trained abroad, mainly in France. Genetic counseling in Switzerland is regulated by the Federal law on genetic testing in humans, GUMG (Bundesversammlung der Schweizerischen Eidgenossenschaft, 2022). No medical degree is required to perform genetic counseling in Switzerland. It is open to a “competent person” (GUMG, § 21 (2)), such as GCs, but reimbursement is generally restricted to medical doctors. As of 2021 there are 43 registered MGs in Switzerland serving a population of 8.7 million citizens.

This study shows how the GC profession can be integrated into clinical genetic services in the German‐speaking region under the current legal framework and what measures and adaptations are necessary in the future to enable GCs to work effectively in inter‐professional teams. We provide a short context of how the GC profession has developed in the German‐speaking region so far and what legal differences we have to deal with in Germany, Austria, and Switzerland.

## 2 METHODS

### 2.1 Study design

#### 2.1.1 Target population participant recruitment

A qualitative research paradigm was considered most appropriate because little is known about this subject area, and it is important to capture the nuances of how students are being integrated into clinics. The methodological framework for data analysis was based on Mayring's inductive content analysis model (2015), as elaborated upon below. For data collection, we utilized a semi‐structured interview guide. Interviews were conducted via telephone or video calls, as outlined in the Appendix S1.

The recruitment process encompassed one clinical geneticist from each student placement institution, directly engaged in supervising the genetic counseling student. Supervisors from all seven placement institutions were contacted by phone and informed in writing about the study objective and the practical procedure. All agreed to participate in the study. The recruitment strategy was specifically designed to target consultants holding elevated hierarchical positions, ensuring their profound understanding of the healthcare systems and financial aspects related to genetic services within their institution and country.

### 2.2 Data collection

The interviews were conducted by phone or video call by the primary investigator using a semi‐structured interview guide. The interview guide contained a catalogue of open‐ended questions developed by the authors that emerged from a review of the international literature on the introduction of the GC profession in other countries and on a survey among scientists and physicians on the involvement of non‐physicians in genetic counseling in Germany (Zerres, 2013). The interviews elicited the expectations and experiences with the student clinical training, the tasks of genetic counseling that the GC could take over from the medical specialist, the degree of independence the students achieved for the different tasks and skills in genetic counseling and the advantages and disadvantages of introducing GCs into interprofessional teams as well as into the healthcare systems.

An inductive approach with open‐ended questions was used to elicit the interviewee's opinions and attitudes. The audio‐recorded interviews took an average of 90 min. Verbatim transcription was performed by the primary researcher. Identifiable information was removed from all transcripts. The text files were anonymized and stored on a password‐protected computer for the time of analysis. All interviews were performed in German. Translation of codes and categories from German to English was performed at the very end of the data analysis.

### 2.3 Data analysis

For data analysis, the methodology centered on employing Mayring's inductive content analysis, a dependable approach that facilitated an exploration driven by the data. This strategy encouraged the extraction of distinctive and potentially divergent viewpoints from the research participants (Mayring, 2015). Transcripts were iteratively coded using the NVivo software (Alfasoft, Frankfurt, Germany) by the first author. These codes were subsequently organized into initial categories that emerged directly from the data. Through iterative examination of the transcripts, recurring categories and subcategories were explored by the first author. To ensure the coherence of codes, categories, and subcategories they were checked by the last author to arrive at a consensus in a personal meeting before they were analyzed, labeled, and assessed by the first author, allowing for the extraction of insightful patterns related to the research questions. In addition, the first and last authors engaged in regular discussions both after analysis and after assessment in personal or digital meetings, respectively.

## 3 RESULTS

### 3.1 Participants

The study included seven medical geneticists, six who identified as female and one male with long‐standing experience as medical genetic specialists. One interviewee was a consultant, three were senior physicians and three others were chief physicians and directors of genetic services.

Following the inductive content analysis, four major categories emerged:

1. Need for GCs in the German‐speaking regions
2. Models of interprofessional teamwork with GCs
3. Legal basis for GC implementation in genetic services
4. Remuneration of GCs

**FIGURE 1:**
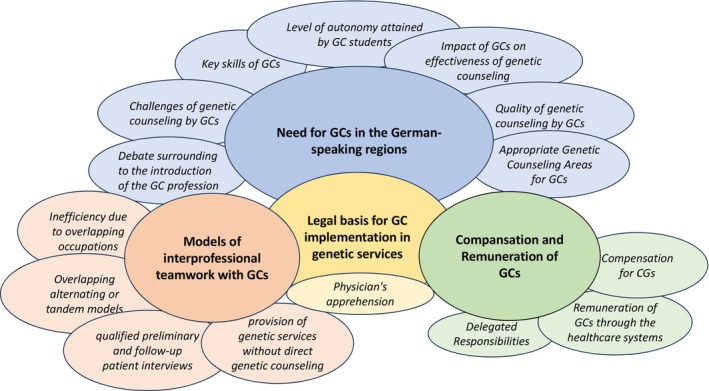
The coding scheme.

The outcome of the analysis comprised a set of primary categories interconnected by a central category, culminating in the creation of a coding schema (see Figure 1).

Due to significant overlap and convergent ideas across all categories offered by the seven study participants, we considered data saturation to be achieved.

#### 3.1.1 Need for GCs in the German‐speaking regions

The question generating the most contentious discussion revolved around the principal necessity of GCs alongside MGs in the German‐speaking regions. This subject gave rise to seven distinct subcategories, each outlining various benefits and drawbacks.

##### Debate surrounding the introduction of the GC profession

The majority favored the incorporation of GCs within healthcare systems, with perceived challenges including apprehensions and concerns related to the unfamiliar scope of the profession, the ambiguous legal framework surrounding GCs, and the absence of proper compensation scales for GCs.

… that was the only [negative] aspect, that you didn't know beforehand what tasks you could transfer and how, what is the job description like in general, but with an open attitude.

I think, we were cautious, but not critical or fearful that anything might not go well or anything might be taken away from the physicians.

Well, that was more of the general consideration of how much do we need to invest to get someone ready to work and whose work time is that actually.

##### Challenges of genetic counseling by GCs

Some MGs mentioned that integrating GC students demanded a significant amount of their time. It was only towards the end of the internship that they became convinced of the potential contributions of GCs within interprofessional teams. Furthermore, there were reservations about the sufficiency of the GCs medical knowledge and concerns that relevant information on rare medical conditions may be missing.

So, I think it could be that the medical background is maybe not there [with the GC], so maybe sometimes an alarm bell doesn't go off that would maybe go off with a doctor. So, I think a great sensitivity and self‐reflective attitude of the GC is needed and also the possibility to ask, am I maybe reaching my limit?

##### Key skills of GCs

When asked which key skills of the GCs had particularly impressed the supervisors, their professional communication techniques and strategies as well as their empathy were mentioned several times.

I think genetic counseling by GCs would be helpful everywhere, because they keep patients in human genetics and have more expertise and also eye for the whole family and for heritability than most other specialists.

I had the impression that this was often a lower‐threshold opportunity for patients to address psychological or social problems than with a doctor. So, from that point of view, we saw a positive aspect there that another quality of consultation comes in.

Patients are more relaxed when the doctors come to them, having experienced [with the GC] before that everything is not so bad.

The [GC], unlike the physicians, have learned a lot more communication skills and have also practiced with test patients. I'm sure they're good at picking up on the patient.

##### Level of autonomy attained by GC students

Following a period of observation that varied in duration (ranging from 5 to 20 consultations), all seven GC students, irrespective of their previous experiences, were proficient, by the conclusion of the 13‐week clincial training, in addressing patient questions. They demonstrated the ability to gather both personal and familial medical histories, create and analyze family pedigrees, perform risk calculations, draft consultation letters and conduct patient interviews autonomously.

We did it in the same way as we do with residents in further training, that the person first runs along and observes, then counsels under supervision and finally does it all themselves.

NN was totally independent [by the end of the internship], … definitely comparable to a first‐year resident.

##### Impact of GCs on effectiveness of genetic counseling

Considering the span of a 13‐week clinical training, a substantial portion of time is naturally allocated to acquainting GC students with the intricacies of organizational processes within genetic services. Additionally, bridging any gaps in knowledge becomes essential, particularly since the participating institutions had no prior experience with such internships. Given this context, it was initially uncertain whether the GC students would be able to offer supplementary services in the 3 months of placement that would alleviate the workload for supervising specialists and participating institutions. What proved even more unexpected was the unanimous consensus among all seven interviewees regarding the prospective integration of GCs into clinical‐genetic consultations. They firmly believed that such an integration would result in a notable increase in the quantity of consultations. When probed about the extent of this anticipated increase, the responses indicated a potential rise of around 50%.

So, by simply having far too few genetic counseling physicians, I really think that both consultation numbers could be increased, waiting times could be decreased, and the quality of care for patients in general could be improved because then there would just be more people who can deliver good quality of counseling.

I think [the waiting time] could definitely be reduced, because the doctors could also do more final consultations, because they only needs half the time. So, we currently calculate 1–1.5 h for an initial consultation. And if maybe half of that is covered by a GC, then we could do twice as many consultations. I could definitely see doing at least half or even more in addition.

##### Quality of genetic counseling by GCs

All interview partners stated that MGs possess a broader scope of medical knowledge and a deeper comprehension of human genetics compared to GCs. According to most interviewees, this distinction might potentially result in reduced quality of genetic counseling if GCs are employed in a manner akin to MGs, without recognizing their unique key competencies. However, these interviewees conceded, that most patients are not well‐versed in medical matters and therefore they might derive greater advantages from the refined counseling skills of a GC than from the extensive medical expertise of a MG.

I don't see the need to always do genetic counseling completely alone. It doesn't do people any favors. … Patients are overwhelmed with information. They don't know what to do with it and can't remember anything when they leave the consultation. And if you then send a four‐page letter from the doctor, it doesn't get any better. You have to know as a GC, what benefit I can provide for this person in their current life situation and psychological distress.

I think the patient would be just as satisfied as they were before. The acceptance would probably increase because they would get a predictable appointment. A GC would probably put even more effort into preparing the family history, which would probably make the patient feel more comfortable. Perhaps more would come up as well, which may not be missed due to lack of time. I could see either a consistent or maybe even an increased quality overall.

##### Appropriate genetic counseling areas for GCs

An assessment of diverse counseling situations and topics during the student internship reveals that, with a couple of exceptions, most students primarily engaged in counseling before genetic analyses were performed and were less frequently involved in the communication of results. In terms of counseling topics, five out of the seven students exhibited a distinct emphasis on familial cancers (ranging from 30% to 90% of their genetic counseling sessions during the students internship), both in the context of diagnostic and predictive genetic testing. Two of the seven students extended their counseling proficiency to encompass other diagnostic queries (ranging from 46% to 70% of their genetic counseling sessions during the students internship), while an additional two students displayed a higher frequency of addressing predictive questions (ranging from 15% to 20% of their genetic counseling sessions during the students internship). In addition to familial cancers, recurring topics of consultation included infertility, recurrent miscarriages, implantation failure, and noninvasive prenatal testing (NIPT).

Some interviewees expressed the belief that, with intensive on‐the‐job training and suitable personal suitability, GCs could feasibly be integrated into nearly all genetic consultations. However, there was consensus that the feasibility decreases with the rarity and complexity of the clinical presentation.

I wouldn't draw any lines there. I think it's all doable, depending on the commitment of the individual. I would transfer everything to [the GC] for the time being.

Genetics is usually not something that's an emergency, so you can sometimes clarify something over the phone with people seeking advice or ask for something again.

Other interviewees were skeptical in this regard and would not assign consultations on clinical genetic questions to a GC:

A dysmorphic child is of course also a challenge for the physician. There I would say, one must have years of experience, that goes then only together. These are perhaps not the topics that I would “offer” someone in the first place. But that is the same with the medical profession. If they're afraid, they can't do it either, and many never can…

#### 3.1.2 Models of interprofessional teamwork with GCs

##### Inefficiency due to overlapping occupations

Given that in Germany and Austria, GCs are not permitted to work autonomously, they must collaborate with MGs with some degree of overlap. This brings forth the question of time management, which elicited varied responses from the interviewees. Three participants indicated that they had already benefitted from the participation of GC students within the counseling team during the student internship, as these students alleviated their workload. On the contrary, four institutions did not witness any workload reduction through the involvement of students; instead, they had to invest more time than the benefits reaped. Nevertheless, these interviewees emphasized their belief that benefits would manifest as the students gained more experience.

Regarding effective collaboration between MGs and GCs within a counseling team, the insights from the student internships gave rise to several suggestions, which can be condensed into three models of interprofessional cooperation.

- overlapping alternating or tandem models,
- qualified preliminary and follow‐up patient interviews and
- provision of genetic (counseling) services without genetic counseling in the legal sense.

##### Overlapping alternating or tandem models

In scenarios employing the alternating or tandem model, the GC takes on the responsibility of patient admission, understanding their expectations, constructing pedigrees, conducting risk calculations, and providing psychological assessments if needed. Furthermore, the GC explains the diagnostic process to the patients. Subsequently, in the tandem model, the responsibility is transitioned to the MG, who, in collaboration with the GC, concludes the genetic counseling process. Conversely, in the alternating model, after delivering medical information and addressing any outstanding queries if necessary, the MG hands back the counseling process to the GC.

I … find it pleasant that I don't have to start with Adam and Eve. I'm only called in when things get really complicated and when that's a challenge. That's when it's fun. Most [physicians] have expressed they need that pedigree survey to get started, to get the feel for the patient. That's something I can't relate to. Maybe everybody is different. Interestingly, none of those who couldn't imagine it have said that anymore, now that we've had this pilot phase with our GCs, because you have a relationship with the GC. You're a team; you know them all at once. In these 5 min of the handover, where you exchange information, you convey a lot and then you also have a feeling for it, prepare the doctor a bit for the situation. In principle, you've gained just as much from that as if you sat there for 20 min and discussed the great‐grandparents.

##### Qualified preliminary and follow‐up patient interviews

One of the internship facilities had a positive experience with autonomous preliminary sessions conducted by the GC through telephone or video conferences. During these sessions, the consultation's objective was deliberated upon, and the family history was gathered. Preliminary findings were also discussed and/or collected, and if necessary, confidentiality releases for relatives were prepared. Additionally, procedural explanations were provided. Consequently, the genetic counseling itself occurred in a succinct manner, under well‐prepared circumstances, in a face‐to‐face discussion involving either just the MG or both the MG and the GC at a later scheduled date. In challenging situations, patients could also find value in follow‐up phone calls or conversations with the GC.

Or sometimes a backtracking, when you call the patients again if they have had an abortion. … That's not a lot of time cost at all, but a lot of times the organization is just too much for you. … A GC could also do that, ask them how they are now, do they want to talk to the doctor again, have they finished the topic of wanting a child or do they want to make another appointment. I'm sure they [the patients] would feel much better cared for.

##### Provision of genetic (counseling) services without genetic counseling in the legal sense

Multiple interviewees highlighted a growing trend where patients are seeking genetic counseling before undergoing diagnostic genetic germline analyses, even though it is not legally mandated, particularly in the context of therapy‐relevant genetic tests (known as companion diagnostics). Considering the escalating number of these consultations and the anticipation of further demand, two interviewees contemplated the possibility of offering qualified medical consultation, rather than genetic counseling in the legal sence, before companion diagnostics. They proposed reserving genetic counseling more for cases with abnormal results. Additionally, the GCs would be responsible for determining the necessity of counseling beyond companion diagnostics. This approach aims to efficiently allocate counseling resources to those with the greatest need and legal requirement, facilitating effective interprofessional collaboration and a rational distribution of available resources.

The models of interprofessional collaboration between GCs and MGs developed in this study differ from other models, as the activities of GCs in German‐speaking countries are restricted by a more or less strict physician reservation and the financing of genetic counseling by GCs is still completely unclear.

#### 3.1.3 Legal basis for GC implementation in genetic services

##### Physician's apprehension

A legitimate concern of all interviewees was the potential violation upon the physician's prerogative in genetic counseling by GCs. Nevertheless, this concern was not deemed an insurmountable barrier by any of the interviewees. In addressing this concern, mention was made of Section 10 (3) of the GenDG, which permits the involvement of non‐medical experts in consultation with the individual's consent. Additionally, reference was drawn to the international legal landscape.

I think you could constructively define the work, who does what and how do we divide that [between physician and GC]. Then, as a physician, you might have more time for the purely medical work. As I said, taking [a] history and [doing] the bureaucratic things, someone else can do that just as well under guidance. That doesn't necessarily need a physician's activity in the consultation.

If this could also be clarified in the English‐speaking world, then it will probably be possible to clarify it somehow in our country as well.

Other interviewees (*n* = 4) were concerned that entrenched practices among physicians might hinder the acceptance of GC. This concerned both a narrow understanding of the physician's prerogative and the traditional division of labor between physicians and other medical staff.

There are always clear rules between doctors and nurses as to which professional group does what with patients. And now there's someone else who also wants to get involved.

The problem is that doctors always look at what they have been doing for years. Taking a medical history, explaining risks, explaining how inheritance works, writing endless letters, ordering a genetic diagnosis. … They don't look ahead, they look back. We have to look ahead. There are now also many therapy options, there is the possibility of getting patients into registries so that they get [an individualized] therapy. That is our medical task, to make preventive recommendations, to actually take clinical action.

The interviewees agreed that the job title *Genetic Counselor* is also a problem for their acceptance of the profession because there is no other translation than ‘Genetischer Berater’ that is reserved for MGs. Other German titles like genetic counseling assistant ‘Beratungsassistent’ do not reflect the academic background of GCs.

A little bit of naming is always important. That's a fundamental problem. If we didn't call it a Genetic Counselor, but a Physician Assistant or a Counseling Assistant, then all the doctors would want one so badly.

#### 3.1.4 Compensation and remuneration of GCs

##### Compensation for CGs

There was considerable uncertainty surrounding the issue of how to fairly compensate GCs within German‐speaking regions. During discussions, interviewees compared the remuneration of GCs to both MGs in training and medical‐technical assistants or physician assistants. All interviewees concurred that the compensation for GCs should notably surpass that of medical technicians or physician assistants. Three interviewees were prepared to provide a salary on par with that of MGs in training for experienced and dedicated GCs.

##### Remuneration of GCs through the healthcare systems

A major source of uncertainty lay in the financial support for prospective GCs, as their contributions currently do not have a designated place within the German, Austrian and Swiss healthcare systems. Consequently, the payment for GCs would either need to be sourced from the revenue generated by MGs when their consulting capacities expand with GC involvement, or alternative funding channels must be identified. However, the latter sources are more likely to be available temporarily, such as through projects or grants, which would offer limited assistance in solidifying the emerging GC profession. Conversely, if GCs were financed through the expansion of MGs' counseling services, concerns arise about potential devaluation of MGs' counseling contributions. While universities can employ GCs as project or other staff members, allowing for a form of cross‐financing, GCs in private practice sectors need to independently earn their salary by billing for their genetic counseling services. Hence, there is a pressing need to establish distinct billing figures for GCs in this context.

##### Delegated responsibilities

A significant impediment to GC funding in Germany is the restriction on delegating personal physician services, as outlined in appendix 24 of the German Bundesmantelvertrag für Ärzte (KBV, 2015). To facilitate effective interprofessional collaboration between MGs and GCs, the medical community in Germany must advocate for the delegation of specific aspects of genetic counseling to GCs and for increasing the number of funded genetic counseling sessions per MG and quarter.

## 4 DISCUSSION

The inaugural MSc program for training GCs in the German language context revealed a significant outcome: following their clinical training, all students could competently execute the distinct parts of a genetic counseling session autonomously, garnering approval from their supervising MGs. Intriguingly, this achievement wasn't limited to students with previous experience in (genetic) counseling or genetic diagnostics before commencing their studies.

The interviews underscored the dual justification for introducing GCs in German‐speaking regions. On the one hand, the need arises from the shortage of available MGs, leading to extended patient waiting times. On the other hand, GCs are anticipated to emphasize crucial competencies in genetic counseling clinics. The expectations extend beyond just enhanced “crisis intervention” during challenging counseling scenarios. A desire for a psychosocially informed approach to conducting consultations was stressed by the interviewees.

A noteworthy advantage of introducing GCs in German‐speaking countries is their potential for sustained commitment to the institutions they serve. Unlike resident physicians who frequently transition to other roles or establish private practices, GCs are more inclined to maintain long‐term associations with their institutions. This enduring relationship can yield a more favorable cost–benefit ratio, as the investment in training and induction of GCs becomes increasingly advantageous over the course of their collaboration.

GCs are not lesser‐qualified or lower‐paid physicians. Rather, they constitute a distinct profession possessing unique communication competencies that partly intersect with those of MGs. Given this differentiation between GCs and MGs, it becomes imperative to both acquire and exhibit the specific key skills of GCs. A pivotal competence of GCs is not centered on reaching a diagnosis, but rather focuses on informing and aiding patients in comprehending diagnostic options and their ensuing implications. This is particularly significant in enhancing the quality of consultations during specific (crisis) scenarios and complex family dynamics. Furthermore, GCs play a role in providing support to patients and their families beyond the immediate consultation – an aspect that has been relatively underutilized within the German‐speaking regions up to this point.

During the clinical training of GC students, the participating human genetics institutions developed diverse collaboration models between MGs and GCs. These models primarily emerged due to the ambiguous legal and financial circumstances surrounding GCs in the German‐speaking countries. Consequently, the implemented tandem and alternating models resemble the mentorship of young resident doctors, who, similar to GCs, are only allowed to work under the guidance of MGs. Conversely, the models involving qualified pre‐ and post‐test consultations, as well as the provision of genetic (counseling) services without genetic counseling by offering qualified medical consultation, rather than genetic counseling in the legal sense before companion diagnostics, showcase how GCs can effectively function within interprofessional teams while adhering to legal regulations.

The primary objective should be to minimize the extent of overlapping activities between MGs and GCs. This is crucial in order to secure funding for the supplementary activities of GCs. Given that independent genetic counseling by GCs is not conceded in Germany and Austria, some level of convergence in the tasks performed by MGs and GCs is, of course, inevitable. These areas of convergence could be effectively utilized for an interactive teach‐back model involving patients. For instance, this approach could add the benefit of enhancing the comprehension of genetic concepts by patients. Importantly, this educational method would achieve these outcomes without making the patients feel lectured or examined (Joseph et al., 2019).

Additionally, the interview‐based study underscores the significant obstacles to the integration of GCs caused by the ambiguous legal context in Germany and Austria, along with the challenge of adequately compensating GCs across German‐speaking nations. Interestingly, the legal frameworks in Switzerland and Germany do not hinder the collaboration of GCs within interprofessional human genetics teams. Switzerland, lacks a doctor's prerogative for genetic counseling, while in Germany, the German Gene Diagnostics Act (§ 10 paragraph 3) allows GCs to collaborate with MGs as “non‐medical experts.” Despite not enabling independent genetic counseling by GCs in Germany, this regulation explicitly permits their active participation.

Even in Austria, which upholds a stringent doctor's prerogative, the law doesn't explicitly exclude GCs. It distinctly encompasses non‐medical practitioners such as psychologists and social workers within the genetic counseling process. Moreover, Austrian legislation includes a dynamic update mechanism through the Gene Technology Book. Functioning as an objective expert opinion, this mechanism offers the flexibility to respond effectively to the rapidly evolving landscape of scientific and technological advancements in human genetics. To establish GCs in Austria, the endorsement of this adaptive approach would necessitate active promotion by the majority of stakeholders, mirroring the successful achievement already observed in Switzerland (Amstad, 2021).

Students in the MSc program at the Medical University of Innsbruck are trained following the international curriculum developed by the EBMG (Skirton et al., 2013). After graduation from an EBMG accredited MSc program and 2 years of professional experience in a genetic service, GCs are able to register as European certified GCs with the EBMG and to carry out genetic counseling in accordance with international standards (Skirton et al., 2017). In the German‐speaking region, GCs work in collaboration with medical geneticists. The extent to which German‐speaking GCs will be able to put their full skill set acquired in their academic training into practice will be dependent on the professional developments over the coming years.

Another significant challenge obstructing the integration of GCs in German‐speaking countries lies in the term “Genetic Counselor” itself. Internationally, this title conveys a level of independence that currently exceeds what is conceivable within the contexts of Germany and Austria. To accurately translate “genetic counselor” into German and emphasize the professional nature of the role, it becomes essential to clarify its academic character. However, any job title incorporating the term “assistant” would undervalue this professional group, placing them to or below a bachelor's level, a situation that must be avoided. The phrase “Genetische:r Fachberater:in” might be a suitable alternative, yet this still needs to be embraced by German‐speaking MGs. The job title genetic counselor gave rise to professional debates not only in non‐English‐speaking European countries (Paneque et al., 2020) but even in the English‐language countries (Means et al., 2020). Therefore we hope that many MSc graduates will register with the EBMG as “European certified genetic counselors” and thus maintain the comparability of the new German professional title “Genetischer Fachberater” with “genetic counselor”. The European Board of Medical Genetics has made great efforts in recent decades to introduce the profession of GCs in genetic services, including title protection, definition of educational and practice standards, and fostering the recognition not only for GCs but also the other two professional groups in the human genetic team, the clinical laboratory geneticists as well as the medical geneticists (Liehr et al., 2017; Paneque et al., 2016, 2017; Skirton, 2017). Some MGs perceive genetic counseling (genetische Beratung) as their distinct and central skill. Interestingly, doctors from other specialties are already authorized to offer genetic counseling in Germany, provided they have undergone additional qualifications.

For an effective and efficient interprofessional collaboration between MGs and GCs in German‐speaking regions, conventional notions of genetic counseling need to be reevaluated. While genetic counseling in the past century was predominantly associated with clinical genetics and the pursuit of descriptive diagnoses (from phenotype to genotype), its scope has now expanded to encompass more varied tasks, not all of which necessitate the involvement of MGs. For instance, the interviewees did not deem it obligatory for patients to undergo genetic counseling with an MG before undergoing a genetic analysis required for therapeutic purposes *and* initiated by a doctor. According to their perspective, consultations preceding the genetic clarification for common queries such as infertility, recurrent miscarriages, familial cancers, non‐invasive prenatal tests (NIPT), and others need not necessarily be conducted by a medical doctor.

However, to achieve this shift, the self‐perception of MGs needs to evolve towards focusing more on tasks that neither other specialists nor GCs can undertake. These include writing and taking responsibility for medical reports, and genetic diagnoses and associating them with personalized therapies, early cancer detection, and prevention as well as giving therapy recommendations. The aspects of guiding patients through family and medical histories, conducting and sharing risk assessments, educating clients about fundamental genetic information, testing options, potential outcomes, and implications, along with offering recommendations based on test results, can be transferred to other professional groups such as GCs who possess the necessary qualifications. Nonetheless, this transformation necessitates a contemporary redefinition of genetic counseling that clearly distinguishes diagnostic activities from counseling.

### 4.1 Study limitations

The study has two main limitations. First, the participation pool was small due to the small number of students involved in the first inaugural German‐taught master's program in Genetic and Genomic Counseling. Additionally, the distribution of participating countries posed a constraint: only one out of the seven students in the initial cohort came from Austria, and none was from Switzerland. Nevertheless, the preponderance of German students in the cohort mirrored the proportional size of Germany among the German‐language countries.

Second, a notable age range variation was observed among the students – spanning from 23 to 55 years at the commencement of the master's program – and the students had diverse prior experiences in medical genetics. All students had undergone prior training in human genetics centers. Three individuals held a PhD degree and had substantial experience as clinical laboratory geneticists. The remaining four students had more limited medical genetic experience, with bachelor's or master's degrees in psychology, healthcare professions, or genetics.

## 5 CONCLUSION

Germany, Austria, and the German‐speaking part of Switzerland have so far not fully implemented the genetic counselor profession. The Medical University of Innsbruck inaugurated the first German‐taught MSc program in Genetic and Genomic Counseling in 2019 leading to the graduation of the first seven German‐speaking GCs in spring 2022. Here we report insights from the students' placement experiences that we used to elucidate models for integrating this profession into pre‐existing medical genetic services across Germany, Austria, and the German‐speaking regions of Switzerland. Depending on the genetic counseling clinic three models have been successfully practiced: (1) the overlapping alternating or tandem model, (2) qualified preliminary and follow‐up patient interviews, and (3) the provision of genetic (counseling) services without genetic counseling in the legal sense. We were able to show that the lack of a legal basis for the work of GCs and the physicians' reservation for genetic counseling are not insurmountable obstacles to the introduction of GCs into existing clinical genetic outpatient clinics in German‐speaking areas. Reliable financing models and a clear definition of the scope of practice in accordance with Middleton, Taverner, Moreton, et al. (2023), Middleton, Taverner, Houghton, et al. (2023) must be established. A clear affiliation with MG‐led centers should help reduce apprehension towards the profession and encourage more support for GCs.

## AUTHOR CONTRIBUTIONS

The primary investigator Simone Heidemann conceptualized and administered the study. She performed all interviews, analyzed, validated, and curated the data, wrote the original draft of the article, and visualized the findings. Gunda Schwaninger supervised the research project in aspects of conceptualization, methodology, data analysis, and validation. As a GC trained in GB, she reviewed the translation of themes and the writing and editing process. Johannes Zschocke gave important intellectual input in the project as well as writing and editing of the manuscript. All authors contributed according to the ICMJE criteria to the manuscript and agree with the publication of the manuscript in the Journal of Genetic Counseling.

## CONFLICT OF INTEREST STATEMENT

Author SH is a graduate of the MSc program in Genetic and Genomic Counseling at the Medical University of Innsbruck. Authors JZ and GS are part of the development team and program director of the master's program in Genetic and Genomic Counseling at the Medical University of Innsbruck.

## ETHICS STATEMENT

Human studies and informed consent: All procedures followed were in accordance with the ethical standards of the Helsinki Declaration of 1975, as revised in 2000 (5). Informed consent was obtained from all interview partners for being included in the study.

Animal Studies: No non‐human animal studies were carried out by the authors for this article.

## Supporting information


Appendix S1

## Data Availability

Authors provide data availability and declare the absence of shared data. The corresponding author confirms that she has full access to all the data in the study and takes responsibility for the integrity of the data and the accuracy of the data analysis. All of the authors gave final approval of this version to be published and agreed to be accountable for all aspects of the work in ensuring that questions related to the accuracy or integrity of any part of the work are appropriately investigated and resolved.
