# Supplementary material for: First experiences with the introduction of genetic counselors in human genetic services in the German‐speaking countries
Source: J Genet Couns. 2024 Nov 11;34(3):e1979. doi: 10.1002/jgc4.1979 (PMC12041831; doi:10.1002/jgc4.1979)
Supplement: Supplementary file 1 — Appendix S1 [file JGC4-34-0-s001.docx]

**Supporting Information**

**Questionnaire guide for the interviews**

**Part I: closed questions regarding characteristics of the** student placement institutions **(selection) to the students**

1. Which kind of facility are you working in? (University, ambulatory health care center, private practice, group practice, others: please specify)
2. How many consultants, residents and office workers are operating in genetic counseling in your facility, in full or part time, respectively?
3. How many genetic counseling did your facility perform altogether per year from 2016 to 2020?
4. What main topics of genetic counseling is your facility focused on? (syndromology, prenatal diagnostics, non-invasive prenatal testing (NIPT), metabolic disorders, hereditary cancers, endocrinology, neuromuscular disorders, epigenetics, ophtalmogenetics, urogenetics, pharmacogenetics, fertility, others: please specify)

**Part II: open questions regarding experiences with the practice and the students (selection) to the interviewees**

1. Which kind of practices did the student perform and which grade of autonomy did she achieved?
2. As far as your experience with the practice goes, which parts of a genetic counseling can be delegated to future genetic counselors in the German-speaking countries and which main topics are most propriate for genetic counselors?
3. Which positive consequences did you expected from the practice, and which one occurred? Were there unexpected positive experiences for your facility?
4. Which negative effects did you suspected from the practice, and which one occurred? Were there unexpected negative effects on your facility?
5. Were there any negative influences on the quality of the genetic counseling due to the inclusion of the genetic counseling students?
6. Did the inclusion of the Genetic counselor student influence the effectivity of the genetic counseling service in your facility and if, how?
7. Which positive consequences do you expect medium-term for the German-speaking genetic counseling services from the inclusion of future genetic counselors in genetic services?
8. Do you suspect negative effects medium-term for the German-speaking genetic counseling services from the inclusion of future genetic counselors in genetic services?
